# Editorial: Characteristics and prevention of the emerging avian influenza A viruses in birds and mammals

**DOI:** 10.3389/fmicb.2025.1551328

**Published:** 2025-02-11

**Authors:** Jinyu Sui, Kaicheng Wang

**Affiliations:** ^1^China Animal Health and Epidemiology Center, Qingdao, China; ^2^Key Laboratory of Animal Biosafty Risk Prevention and Control (South), Ministry of Agriculture and Rural Affairs, Qingdao, China; ^3^Key Laboratory of Animal Biosafty, Qingdao, China

**Keywords:** avian influenza, cross-species transmission, zoonotic diseases, characteristics, prevention

Avian influenza A virus (AIV) has circulated widely in domestic poultry and wild birds. Especially since 2021, the highly pathogenic avian influenza (HPAI) H5 virus has led to mass deaths and slaughter of poultry, peaking at 146 million in 2022 (WOAH, 2024a), and the wild bird HPAI cases have had the widest geographical spread in 76 countries and territories around the world, with 101,817 wild bird cases, a historically unprecedented occurrence (WOAH, 2024b). Additionally, the HPAI H5 virus has increasingly crossed the species barrier, and more than 70 mammalian species infected with the H5 virus have been reported to the World Organisation for Animal Health (WOAH) with over 1,100 cumulative outbreak records since 2021, including marine mammals, terrestrial carnivorous and scavenging mammals, livestock, and domestic cats and dogs (WOAH, 2025). In 2024, an unprecedented outbreak of HPAI H5N1 in dairy herds occurred in the USA; by November 2025, 928 infected dairy herds had been confirmed in 16 states, with the virus spread within and between herds, infections in poultry and cats, and spillover into humans (Figure 1) (US CDC, 2025). Meanwhile, with the increasing number of infected mammalian species, mammal-to-mammal transmission may have occurred during the outbreaks of H5N1 viruses in minks, sea lions, and cows (Eisfeld et al., 2024). This trend is particularly concerning as it indicates that the viruses may be evolving to adapt more effectively to mammalian hosts.

**Figure 1:**
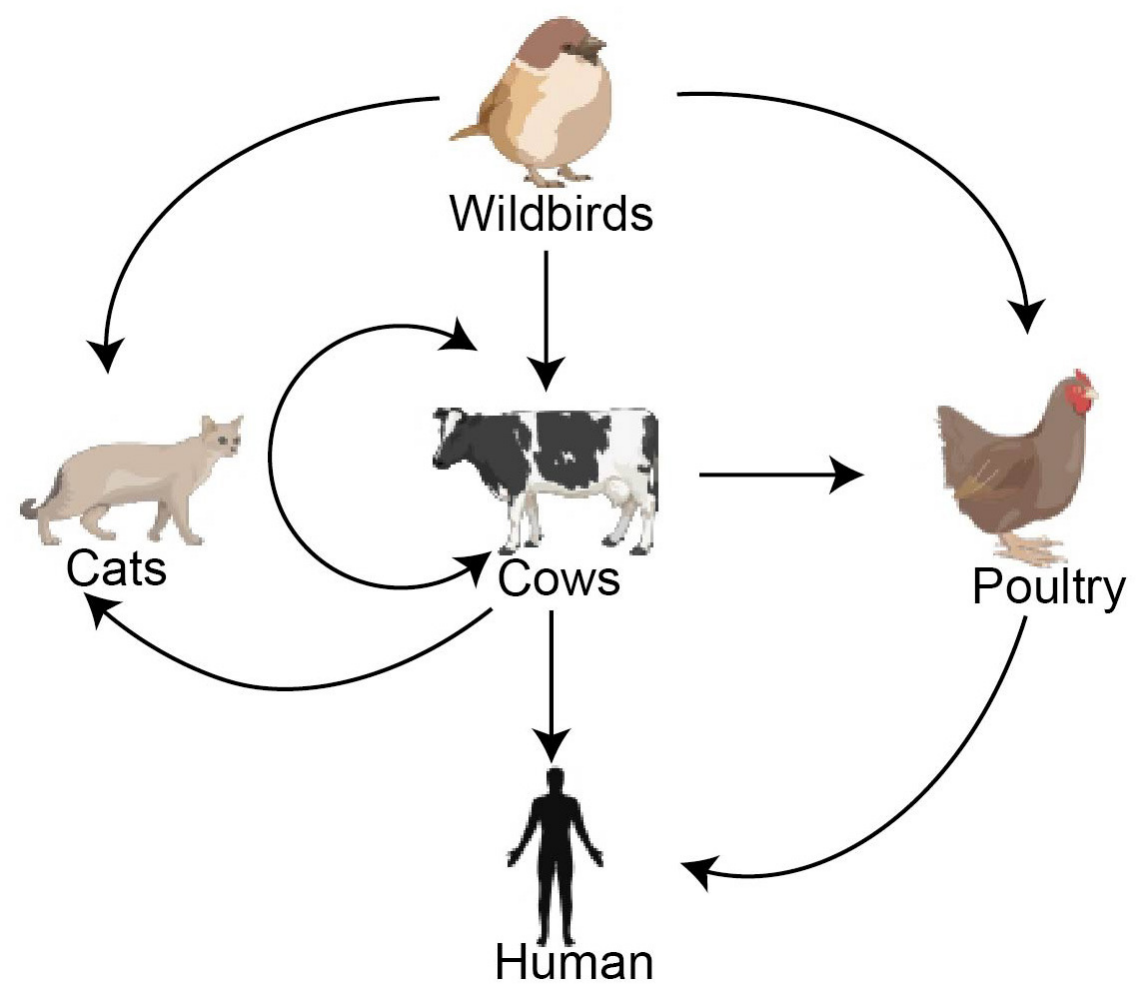
Virus transmission during the HPAI H5N1 outbreak in dairy cows in the USA.

The ongoing epidemics of AIV in poultry and mammals pose an increased risk of spillover infections to humans. According to the latest statistics from the World Health Organization (WHO, 2018, 2024), since January 2003, there have been 939 cases of H5N1 infection in humans, including 464 deaths, which equates to a case fatality rate (CFR) of 49%, and since early 2013, a total of 1568 confirmed human infections with H7N9 viruses have been reported, including 616 fatal cases (CFR: 39%). In 2024, 40 human cases associated with exposure to sick dairy cows had been reported to be infected with HPAI H5 avian influenza viruses (US CDC, 2025). The primary risk factor for human infection is exposure to infected live or dead birds or contaminated environments. In contrast, the CFR was much higher in those with a history of exposure to sick and dead birds compared to the other exposure groups, which may be related to the higher viral loads carried by the sick and dead birds (Li F. et al.).

The HAs of AIV strains show a preference for α2,3-linked sialic acids (SA-α2,3-Gal), whereas the HAs of human influenza virus strains bind preferentially to α2,6-linked sialic acids (SA-α2,6-Gal). Human tracheal epithelium expresses mainly SA-α2,6-Gal and alveoli and bronchioles express mainly SA-α2,3-Gal. Consequently, severe pneumonia is the common lesion in human cases with confirmed H5N1 virus infection (Van Riel et al., 2007). Notably, the bovine H5N1 virus was found to bind to SA-α2,6-Gal in human upper airways while still exhibiting a strong preference for avian-like SA-α2,3-Gal. In addition, the significant binding of the bovine H5 virus to tissues such as the conjunctiva, bronchioles, and lung is consistent with the reported clinical symptoms in humans, which include conjunctivitis and mild respiratory symptoms (Garg et al., 2024; Song et al., 2025).

The receptor-binding properties of hemagglutinin (HA) are critical for influenza A virus–host adaptation. Mutations in the receptor-binding region of HA can alter binding properties, leading to shifts in receptor preference and influencing interspecies transmission. Polymerase basic protein 2 (PB2) is an important component of the AIV RNA polymerase and plays a critical role in the replication and transcription of the viral genes. Mutations of the specific amino acid residues in the PB2 protein can enhance replication efficiency and adaptability to mammalian hosts. The sequences of AIVs from the human and mammalian cases had key molecular markers associated with mammalian adaptation and pathogenicity, particularly in the HA and PB2 gene segments. For example, HA T192I, PB2 Q591K, D701N, and E627K in the H5N1 virus (FAO/WHO/WOAH, 2024); HA G186V and Q226L/I, PB2 E627K, D701N, and K702R in the H7N9 viruses, (WHO, 2018); and HA Q226L, PB2 E627K, and D701N in the H9N2 viruses (Tan et al.). The three mutations, namely PB2 E627K, HA E186D, and HA Q222H, which are potentially associated with enhanced virulence and human adaptation, were identified in the genome of a Canadian case who presented with conjunctivitis and fever and progressed to respiratory failure (Jassem et al., 2024). The PB2 E627K mutation was also identified in the genome of the human case with exposure to infected dairy cows (FAO/WHO/WOAH, 2024). Although the species barrier largely restricts the cross-species transmission of AIVs, these mutations increase the binding ability with SA-α2,6-Gal and facilitate mammalian adaptation.

To date, AIV strains detected in humans and mammals have not established adaptations to mammalian hosts or acquired the capacity for sustained human-to-human transmission. All reported human cases have occurred after exposure to infected animals or contaminated environments. Based on the information reported, the joint assessment by the Food and Agriculture Organization of the United Nations (FAO)-WHO-WOAH concludes that the global public health risk of HPAI H5/H7 viruses remains low (FAO/WHO/WOAH, 2024).

However, HPAIV remains a significant global challenge due to its widespread circulation and the dramatic increase in spillovers between wild birds and mammals. Several preventive measures against AIVs are required for averting potential future influenza pandemics, including Improved global surveillance of wild birds, poultry, mammals, and humans is central to informing the threat of HPAIVs. Fair et al. present a series of recommendations to strengthen wild bird surveillance through a more connected network. Vaccination is an important strategy for the control of HPAIVs, and it is essential to continue evaluating candidate vaccine viruses in order to prepare for pandemics. A study proposes a novel computational method for predicting antigenic distances, which exhibits low error in virus antigenicity prediction and achieves superior accuracy in discerning antigenic drift (Li X. et al.). The constantly evolving genome of AIV poses a challenge for effective antiviral therapeutics due to mutations in the viruses, particularly those on the surface protein such as HA and neuraminidase (NA). Previous studies revealed that double or triple combinations of antiviral drugs or antibodies contribute to synergistic antiviral activity against a panel of AIVs (Hoang et al.).

The contributions in this Research Topic underscore the characteristics and prevention of HPAIV in birds and mammals. It is recommended that future research focus on additional studies or surveillance to contain the elevated public health risk posed by the widespread distribution and ongoing evolution of AIVs.

## References

[B1] Eisfeld A. J. Biswas A. Guan L. Gu C. Maemura T. Trifkovic S.. (2024). Pathogenicity and transmissibility of bovine H5N1 influenza virus. Nature 633, 426–432. 10.1038/s41586-024-07766-6 38977017 PMC11390473

[B2] FAO/WHO/WOAH (2024). Updated joint FAO/WHO/WOAH assessment of recent influenza A(H5N1) virus events in animals and people. Available at: https://www.who.int/publications/m/item/updated-joint-fao-who-woah-assessment-of-recent-influenza-a(h5n1)-virus-events-in-animals-and-people (accessed December 6, 2024).

[B3] Garg S. Reinhart K. Couture A. Kniss K. Davis C. T. Kirby M. K.. (2024). Highly pathogenic avian influenza A(H5N1) virus infections in humans. N. Engl. J. Med. 24:610. 10.1056/NEJMoa2414610 39740051

[B4] Jassem A. N. Roberts A. Tyson J. Zlosnik J. E. A. Russell S. L. Caleta J. M.. (2024). Critical illness in an adolescent with influenza A(H5N1) virus infection. N. Engl. J. Med. 24:890. 10.1056/NEJMc2415890 39740022

[B5] Song H. Hao T. Han P. Wang H. Zhang X. Li X.. (2025). Receptor binding, structure, and tissue tropism of cattle-infecting H5N1 avian influenza virus hemagglutinin. Cell 22:19. 10.1016/j.cell.2025.01.019 39848246

[B6] US CDC (2025). H5 Bird Flu: Current Situation. Available at: https://www.cdc.gov/bird-flu/situation-summary/index.html (accessed January 10, 2025).

[B7] Van Riel D. Munster V. J. de Wit E. Rimmelzwaan G. F. Fouchier R. A. Osterhaus A. D.. (2007). Human and avian influenza viruses target different cells in the lower respiratory tract of humans and other mammals. Am. J. Pathol. 171, 1215–1223. 10.2353/ajpath.2007.070248 17717141 PMC1988871

[B8] WHO (2018). Analysis of recent scientific information on avian influenza A(H7N9) virus. Available at: https://www.who.int/publications/m/item/analysis-of-recent-scientific-information-on-avian-influenza-a(h7n9)-virus (accessed December 6, 2024).

[B9] WHO (2024). Avian Influenza Weekly Update Number 975. Available at: https://cdn.who.int/media/docs/default-source/wpro—documents/emergency/surveillance/avian-influenza/ai_20241129.pdf?sfvrsn=5bc7c406_51 (accessed December 6, 2024).

[B10] WOAH (2024a). High Pathogenicity Avian Influenza (HPAI) – Situation Report 66. Available at: https://www.woah.org/en/document/high-pathogenicity-avian-influenza-hpai-situation-report-66/ (accessed January 23 2025).

[B11] WOAH (2024b). Strategic challenges in the global control of high pathogenicity avian influenza. Available at: https://doc.woah.org/dyn/portal/index.xhtml?page=aloandaloId=44448 (accessed January 23, 2025). 10.20506/rst.SE.3563 39713829

[B12] WOAH (2025). Cases of avian influenza in mammals. Available: https://www.woah.org/en/disease/avian-influenza/#ui-id-2 (accessed January 23, 2025).

